# Anodal Transcranial Direct Current Stimulation Increases Bilateral Directed Brain Connectivity during Motor-Imagery Based Brain-Computer Interface Control

**DOI:** 10.3389/fnins.2017.00691

**Published:** 2017-12-07

**Authors:** Bryan S. Baxter, Bradley J. Edelman, Abbas Sohrabpour, Bin He

**Affiliations:** ^1^Department of Biomedical Engineering, University of Minnesota, Minneapolis, MN, United States; ^2^Institute for Engineering in Medicine, University of Minnesota, Minneapolis, MN, United States

**Keywords:** brain-computer interface, transcranial direct current stimulation, connectivity, BCI, tDCS

## Abstract

Transcranial direct current stimulation (tDCS) has been shown to affect motor and cognitive task performance and learning when applied to brain areas involved in the task. Targeted stimulation has also been found to alter connectivity within the stimulated hemisphere during rest. However, the connectivity effect of the interaction of endogenous task specific activity and targeted stimulation is unclear. This study examined the aftereffects of concurrent anodal high-definition tDCS over the left sensorimotor cortex with motor network connectivity during a one-dimensional EEG based sensorimotor rhythm brain-computer interface (SMR-BCI) task. Directed connectivity following anodal tDCS illustrates altered connections bilaterally between frontal and parietal regions, and these alterations occur in a task specific manner; connections between similar cortical regions are altered differentially during left and right imagination trials. During right-hand imagination following anodal tDCS, there was an increase in outflow from the left premotor cortex (PMC) to multiple regions bilaterally in the motor network and increased inflow to the stimulated sensorimotor cortex from the ipsilateral PMC and contralateral sensorimotor cortex. During left-hand imagination following anodal tDCS, there was increased outflow from the stimulated sensorimotor cortex to regions across the motor network. Significant correlations between connectivity and the behavioral measures of total correct trials and time-to-hit (TTH) correct trials were also found, specifically that the input to the left PMC correlated with decreased right hand imagination performance and that flow from the ipsilateral posterior parietal cortex (PPC) to midline sensorimotor cortex correlated with improved performance for both right and left hand imagination. These results indicate that tDCS interacts with task-specific endogenous activity to alter directed connectivity during SMR-BCI. In order to predict and maximize the targeted effect of tDCS, the interaction of stimulation with the dynamics of endogenous activity needs to be examined comprehensively and understood.

## Introduction

### Transcranial direct current stimulation

Transcranial direct current stimulation (tDCS) of the human brain has been increasingly investigated with the resurgence of research into the effects of noninvasive electrical brain stimulation in the early 2000s (Nitsche and Paulus, 2000; Johnson et al., 2013; Paulus and Opitz, 2013; Bestmann et al., 2015). tDCS consists of injecting a low level of current (generally <2 mA) into the head of a subject through multiple electrodes located on the scalp or extracephalically. Modeling studies using both standard two-electrode and multi-electrode configurations have found that current reaches the cortex, and depending on electrode configuration, deeper brain structures, with levels that have been shown *in vitro* to affect the potential of spontaneous neuronal firing (Bikson et al., 2004; Sadleir et al., 2010; Kabakov et al., 2012; Kuo et al., 2013; Opitz et al., 2016). These neuronal effects most likely stem from a variety of sources including membrane depolarization and hyperpolarization of the dendrites and axons of pyramidal cells as well as secondary effects on membrane resistance (Stagg and Nitsche, 2011; Paulus and Rothwell, 2016). Behaviorally, the effects of tDCS on the motor system have been found to affect motor performance and learning when the motor network is stimulated (Reis et al., 2009; Reis and Fritsch, 2011; Buch et al., 2017). A variety of electrophysiological, hemodynamic, and spectroscopic methods have been used to investigate alterations of neural activity from tDCS including increases in the BOLD signal and alterations in excitatory and inhibitory neurotransmitter balance (Jang et al., 2009; Stagg et al., 2009; Antal et al., 2011; Zaehle et al., 2011; Jog et al., 2016; Muthalib et al., 2017). The *in vivo* effects of tDCS on endogenous resting and task specific brain oscillations is less well-understood, and has only recently begun to be investigated with EEG, MEG, and invasive recordings (Soekadar et al., 2013, 2014; Notturno et al., 2014; Roy et al., 2014; Bergmann et al., 2016; Opitz et al., 2016; Krause et al., 2017).

An emerging hypothesis relating the effect of noninvasive neuromodulation to brain activity utilizes a long-term potentiation rationale for targeting brain areas that are specifically active during a task or rest (Bikson and Rahman, 2013). Fox et al. examined the effects of transcranial magnetic stimulation (TMS) targeting and found specifically that if the areas targeted overlapped with correlated or anti-correlated resting state networks, as determined by fMRI, there was an effect on neurological symptoms in patients (Fox et al., 2014). Further work using a similar approach for targeting resting state activity in the motor network with tDCS found an increase in excitability with anodal stimulation of correlated areas as compared to anodal-cathodal stimulation of anti-correlated areas (Fisher et al., 2017). Concurrent stimulation of involved areas during motor performance and learning has specifically led to improvements in performance compared to stimulation prior to, or after, task performance (Buch et al., 2017). Our group recently found a decrease in time to hit and an increase in EEG alpha and beta band power following simultaneous tDCS over the sensorimotor cortex during motor imagery based EEG brain-computer interface (BCI) performance (Baxter et al., 2016).

### The motor system and noninvasive brain-computer interfaces

The development of noninvasive BCI has allowed individuals with motor dysfunctions to control computers and devices in the lab (Wolpaw et al., 1991; Mak and Wolpaw, 2009; Millán et al., 2010; He et al., 2013, 2015; Scherer and Pfurtscheller, 2013; Yuan and He, 2014) and in the home (Sellers et al., 2009) in real-time using self-modulated brain rhythms or external stimuli. A predominant paradigm for continuous control of an output device is using motor imagination (MI) with sensorimotor rhythm modulation. In order to voluntarily modulate their sensorimotor rhythms, subjects kinesthetically imagine moving a body part without executing the movement. This imagination engages similar networks to motor execution (ME) and generates an event related desynchronization in alpha (8–13 Hz) or beta (15–30 Hz) frequencies, corresponding to a local decrease in power (Pfurtscheller and Lopes da Silva, 1999; Lotze and Halsband, 2006), in the sensorimotor cortical areas responsible for controlling the body part. An event-related synchronization also may occur in contralateral sensorimotor regions. A recent meta-analysis of fMRI studies found premotor (PMC) and somatosensory (S1) regions predominantly active during MI as well as more distributed areas in the frontal and parietal cortices, including the inferior frontal gyrus, supplementary motor area (SMA), primary motor cortex (M1), and superior parietal cortex (PC) (Hétu et al., 2013). While fMRI yields precise localization of an indirect measure of neuronal activity, the temporal resolution is on the order of seconds which does not allow an examination of most oscillatory dynamics.

Regions of the motor network are thought to be responsible for similar tasks during both ME and MI, though there are some known differences in network activity between these two cognitive actions. The PMC is involved in both execution and imagination though different sub-domains are active in each. The PMC is left hemisphere lateralized and is used for motor planning and selection, including selecting the hand to perform a unimanual task (Rushworth et al., 2003). The dorsal PMC also bilaterally increases in activation for contralateral hand execution. For MI, the dorsal and ventral PMC are specifically active (Lotze and Halsband, 2006). The pre- and post-SMA is involved in motor movement preparation, initiation, and execution (Lotze and Halsband, 2006). For MI, the posterior regions of the SMA are specifically active. The sensorimotor cortex (SMC) is involved in both ME and kinesthetic imagination but the degree of activation may depend on the complexity of the imagery movement (Lotze and Halsband, 2006). The contralateral primary motor cortex is active during ME with the contralateral S1 cortex activated with sensory feedback, such as the feeling of pressure on the hand when closing your fingers into a fist. In addition to this activation, there is inhibition from the activated hemisphere to motor cortex ipsilateral to the executed hand movement. The PC, and specifically the PPC is involved in motor preparation and attention as well as visual motor transformation and performing visuo-motor tasks (Andersen and Buneo, 2002; Rushworth et al., 2003). During motor imagery, orienting visual attention with or without arm movements leads to BOLD increase in differing areas of the PC; these areas may be somewhat lateralized to the left hemisphere (Rushworth et al., 2001). During BCI performance, subjects likely direct visual attention and eye movement to the target and to the cursor during the feedback phase, though this may not be the case when subjects are solely performing MI. In addition to these cortical areas, the cerebellum and subcortical regions are also involved in both ME and MI (Lotze et al., 1999; Lotze and Halsband, 2006).

### Source imaging and BCIs

The temporal resolution of EEG is on the order of milliseconds but standard analysis of EEG data on the sensor level does not allow for high spatial resolution. Source imaging, which involves solving the inverse problem of mapping EEG sensor activity to the brain using Maxwell's equations and the physical properties of head tissues, has been developed over the last few decades (He et al., 1987, 2011b; Hämäläinen and Sarvas, 1989). Based on the specific algorithm for performing this transformation, modeling and event-related potential studies have found localization errors of 7 mm or less (Michel et al., 2004; Im et al., 2007). Source imaging analysis of MI both without and with feedback has been demonstrated to have higher signal-to-noise ratio than sensor data, which can lead to improved MI classification (Qin et al., 2004; Kamousi et al., 2005, 2007; Cincotti et al., 2008; Yuan et al., 2008; Edelman et al., 2016). While source based analysis allows us to examine how brain areas are active over time, more explicit analysis of the interaction of different brain areas are needed to functionally understand how information flows within the network.

### Connectivity

There are multiple families of methods that have been used to examine undirected and directed connectivity during cognitive tasks following tDCS using both direct electrophysiological and indirect hemodynamic measurements (Meinzer et al., 2012; Luft et al., 2014). The two activity based classes of connectivity are directed connectivity and functional connectivity; the former is measured via causal directional relationships between two time series, while the latter is measured as a correlation or anti-correlation between two time series (Kaminski and Blinowska, 1991; Baccalá and Sameshima, 2001; Babiloni et al., 2005; Astolfi et al., 2007; He et al., 2011a; Friston et al., 2013).

#### Connectivity in the motor system

The connectivity of networks underlying ME and MI has been studied extensively. The unilateral left and right hand ME and motor imagery networks have been compared by applying directed connectivity to fMRI data (Gao et al., 2011). Gao and colleagues reported stronger connectivity amongst the motor network in ME than MI. They found significant intrahemispheric connections within the contralateral hemisphere and between the contralateral and ipsilateral PMC and PPC. Anwar and colleagues used multimodal imaging to examine effective and functional connectivity across the motor network during right-hand finger movement task performance while recording with multiple modalities including fMRI, fNIRS, sensor-based EEG, and source-based EEG and found bidirectional connections between the right dorsolateral prefrontal cortex (DLPFC), right PMC and right SMC (Anwar et al., 2016). Importantly, they found source-based EEG analysis to have the greatest unidirectional flow from SMC to PMC, SMC to DLPFC, and PMC to DLPFC. Other studies have found that PPC is connected to the posterior SMA and PMC (Rizzolatti et al., 1998; Lotze and Halsband, 2006; Davare et al., 2010). Frequency specific connectivity during MI have also been evaluated, though these analyses have generally been confined to the SMC (Kuś et al., 2006; Hamedi et al., 2016). These works examined time and frequency based measures such as Granger causality and coherence metrics and found connections both unilaterally and bilaterally between the SMC and frontal areas during MI. Specific to the BCI context, Billinger and colleagues investigated single trial offline task classification using sensor and source based connectivity measures and found no improvement over power and frequency based features for either sensor or source based analysis (Billinger et al., 2013).

The functionality of these connections has also been investigated through a variety of modalities. The PMC is directly connected to the primary motor cortex; using TMS, the PMC has been found to influence the primary motor cortex during ME depending on parameters of the task, including force delivered and precision of movements, and that this connection is inhibitory during rest (Grafton, 2010; Davare et al., 2011). Connections from the SMA to SMC and PMC inhibit movement execution during MI (Kasess et al., 2008). The function of interhemispheric connections across the corpus callosum between bilateral regions, either inhibitory, excitatory, or both, is an ongoing debate in literature (van der Knaap and van der Ham, 2011).

#### Connectivity and tDCS

Connectivity analysis using EEG following transcranial current stimulation was initially performed by Polania et al. who found that after the application of anodal tDCS during rest there was an increase in undirected intrahemispheric connectivity in the alpha, beta and high gamma frequencies near the stimulating electrode, and decreased interhemispheric connectivity in the alpha and beta bands, both during motor task performance (Polanía et al., 2011). Further studies examining effects on the motor network also found brain state dependent effects following stimulation. Feurra and colleagues found an increase in TMS MEP amplitude during MI following resting state anodal tDCS of the right PC, whereas this effect was not present during motor action observation (Feurra et al., 2011). Notturno and colleagues examined EEG functional connectivity using coherence and found no difference between anodal and sham stimulation of the motor cortex during motor movement, but altered coherence during rest (Notturno et al., 2014). Polania et al. also examined cortical-subcortical connections with fMRI and found increased functional connectivity between the left primary motor cortex (M1) and the ipsilateral thalamus and caudate nucleus following anodal stimulation (Polanía et al., 2012). Sehm et al. found functional connectivity effects on the resting state network during and after anodal stimulation of unilateral and bilateral primary S1 (Sehm et al., 2012). Holland et al. examined effective connectivity during a visual object naming task within the left frontal cortex using DCM on concurrent tDCS-fMRI of the inferior frontal cortex. They found a stronger negative backward connection from inferior frontal sulcus to ventral PMC during anodal stimulation compared to sham indicating stronger inhibition from IFS to vPMC, and behaviorally found improved reaction time. Further work examining both task specific (Meinzer et al., 2012) and resting state has shown similar effects due to anodal stimulation (Keeser et al., 2011; Peña-Gómez et al., 2012; Amadi et al., 2014). Combined, these results suggest anodal stimulation increases connectivity near the stimulation electrode as well as to more distant sites intra- and interhemispherically, though the specific effects and regions affected are dependent on the task being performed, the networks involved in the task, and the regions connected to the stimulated area.

### Motivation

Sensorimotor rhythm-based BCI is a useful experimental technology to evaluate the interaction of stimulation and endogenous event-related oscillations as unilateral hand imaginations yield different bilateral signals generated by the sensorimotor cortex. Previously we reported changes in performance and localized alpha and beta band power following anodal stimulation (Baxter et al., 2016). The aims of this study were two-fold: (1) to determine connectivity changes during sensorimotor rhythm-based BCI control following simultaneous anodal high-definition (HD)-tDCS of the sensorimotor cortex, and (2) to examine correlations between behavioral metrics and connectivity patterns within the motor imagery network. We utilize HD-tDCS as, based on theory and simulation studies, the current is confined to a smaller region of the brain compared to conventional tDCS, allowing for more precise localization of the effect of stimulation (Dmochowski et al., 2011; Kuo et al., 2013). This improved localization allows us to better understand the effect of local stimulation on both nearby and remote areas, as well as the interconnections of these areas.

We analyzed data recorded during sensorimotor rhythm BCI performance while subjects controlled a moving cursor on the screen prior to and following anodal stimulation of the left sensorimotor cortex. We used a data-driven approach to determine regions-of-interest during BCI control across the cortex, calculated the connectivity between these regions, and determined the changes that resulted from the tDCS. We found alterations in the connectivity of the network based on the laterality of the hand imagination, with a greater number of changes in connectivity during right hand imagination. In addition, we examined the relationship between performance and connectivity measures and found both significant positive and negative correlations between specific connections and performance measures. By combining analyses of connectivity changes after stimulation and the correlations of connectivity values with performance, we aim to inform the functional targeting of networks of interest to maximize stimulation effects and develop multifocal closed loop-noninvasive stimulation on a subject specific level.

## Materials and methods

### Experimental setup

Twelve right-handed healthy subjects (8 female) naive to motor imagery (MI) BCI control participated in these experiments (Age: 19–39 years; Mean: 23.58 years; SD: 4.97 years). Subjects were randomly assigned to either Anode or Sham stimulation groups. Included subjects had >62.5% mean accuracy and were considered to have competent control of the BCI (Anode: 72 ± 2% and Sham: 69 ± 2%; mean ± standard error). All procedures and protocols were approved by the University of Minnesota Institutional Review Board.

A 64-channel Biosemi EEG cap with active electrodes and an ActiveTwo amplifier were used to record the EEG signal at 1024 Hz (BioSemi, Amsterdam, Netherlands). A tDCS device with a high-definition (4 × 1) tDCS adapter was used in a Laplacian configuration to deliver 2 mA of current to the center electrode with four return electrodes (Soterix Medical, NY, USA). Conductive gel (Signa Gel, Cortech Solutions) was applied to reduce electrode offsets to below 30 mV for EEG electrodes and impedances under 1 kΩ for tDCS electrodes. The EEG cap was adapted to fit HD-tDCS electrodes adjacent to EEG electrodes arranged according to the international 10/20 system. The center electrode (anode) was placed between C3/CP3 and surround electrodes (cathodes) were placed between CP3/P3, C1/FC1, C5/FC5, and C3/FC3 at a radius of 3.5 cm from the center electrode. For the Anodal group, stimulation consisted of 20 min of 2 mA stimulation with a 30 s ramp up at the start of stimulation and a 30 s ramp down at the end. For the Sham group, for stimulation, the tDCS device ramped up and down over approximately 45 s at the beginning and end of the 20 min.

Subjects were seated in a chair 90 cm from an LCD monitor where experimental stimuli were displayed. Subjects were instructed to remain still during the experimental trials. BCI2000 software was used to present experimental stimuli and record EEG data. Subjects were instructed to kinesthetically imagine opening and closing their respective hand unilaterally based on the target location. The trial structure consisted of a baseline rest period (3 s), planning phase (3 s), and online performance (6 s maximum). Subjects performed 72 trials of the left/right BCI task before stimulation (Prestim); the first 18 trials were removed as at the start of each session the normalizer, embedded in the software, needed to adjust for the subject and session. Following this, the tDCS system was turned on and stimulation was started. During stimulation, subjects performed 90–108 trials depending on individual resting time between runs. The tDCS device was then turned off and the subject immediately performed 72 trials (Post_0_), followed by a visual oddball task for 13 min to engage the subject in a controlled task, while allowing a rest from the BCI task. Finally, subjects performed 72 trials during the delayed time period from approximately 25 to 37 min post stimulation (Post_25_). Subjects participated in three sessions with the time between sessions at least 48 h.

The control system used the autoregressive filter implemented in BCI2000 (Schalk et al., 2004) to estimate the 11–13 Hz power at the C3/C4 electrodes before and after stimulation to control the cursor during the BCI task. During stimulation, C3 was usually affected by stimulation artifacts and was removed on a session by session basis; in these circumstances a surrounding electrode that was not affected by stimulation artifacts was used instead (see **Figure 2** for example EEG traces of control electrodes). The control signal was calculated based on a linear classifier with inputs composed of the positively weighted power in C4 and the negatively weighted power in C3. A normalizer was used with the classifier to reduce any directional bias in the cursor movement due to a subject's difference in relative power between C3 and C4. After each trial, the normalizer removed the offset by subtracting the mean and scaling the classifier output to unit variance based on the weighted sum of C3 and C4 during the online period of the preceding 30 s. For further details see (Baxter et al., 2016).

### Behavioral measures

The time-to-hit (TTH) behavioral metric is the time from the beginning of the feedback period of a trial to the time the cursor hits the target; subjects had a maximum of 6 s to hit the target. The total correct behavioral metric is the total number of correct trials in each block. Both metrics were divided into right and left hand trials due to previous results suggesting there are directional effects of stimulation (Baxter et al., 2016).

### Signal processing

Raw data was high pass filtered within hardware at 1 Hz and notch filtered at 60 Hz. Offline processing was performed using custom scripts utilizing the EEGLAB toolbox (Delorme and Makeig, 2004) in Matlab (The Mathworks, Inc., MA, USA). Data was low pass filtered at 110 Hz and the mean of each channel was removed. Electrodes were referenced to the common average and downsampled to 250 Hz. Independent Component Analysis (fast-ICA) (Hyvarinen, 1999) was run on concatenated data from all non-stimulation blocks for each session. Components corresponding to eye movement, eye blink, and muscle artifact were removed (Jung et al., 2000). We visually examined the EEG time course data and removed electrodes that displayed a drift from their mean over time and spherically interpolated their activity (Delorme and Makeig, 2004); these were primarily prefrontal or temporal electrodes. Those trials that were contaminated with artifacts during baseline or task performance, respectively, not removed by ICA were discarded. Following removal and interpolation of bad channels, and removal of trials with significant artifactual activity, channels were rereferened to the common average and channel means were removed.

For the mean baseline values used to characterize the noise for source imaging, we included all clean trials remaining after artifact rejection and preprocessing. The 1 s prior to the appearance of the target, during the inter-trial interval, was used as the baseline. For analysis, we removed the first 500 ms of the trial, as there was frequently an ERP artifact due to the cursor appearance, as well as the final 250 ms of the trial, as there was frequently an additional artifact. The data within the 500 ms time window that contained the largest power difference was then used for the analysis of the online data. The time courses were detrended and standardized prior to model fitting and further analysis.

### Source analysis

The BEM forward model was calculated using OPENMEEG (Gramfort et al., 2010) with relative conductivity values of Skin/Skull/Brain: (1/0.0125/1) using a quasistatic approximation mapping 64 electrodes to 15,002 dipoles covering the entire cortical surface. A common head model based on the Colin27 head was used for all source analysis with electrodes located in the Biosemi 64 channel EEG configuration. *M* = *GD*, with M indicating the EEG sensor measured values, G indicating the gain matrix from the forward problem mapping of noiseless data from the dipole sources to the sensors, and D indicating the dipole current source density. As this is an underdetermined problem, we employed Tikhonov regularization with the weighted minimum norm approach to estimate the dipole current density distribution using Brainstorm (Lawson and Hanson, 1987; Hämäläinen and Ilmoniemi, 1994; Tadel et al., 2011). The weighted minimum norm solution is given by

$$\hat{D}={\left(W^{T}W\right)}^{-1}G^{T}{\left(G{\left(W^{T}W\right)}^{-1}G^{T}+\lambda I\right)}^{-1}M$$

where $\hat{D}$ is the estimated dipole cortical current density (CCD), *W* is the weight matrix, λ is the regularization parameter, and *I* is the identity matrix (Grech et al., 2008). Where *W* = Ω ⊗ *I* with ⊗ denoting the Kronecker product and Ω being the norm of the columns of *G*.

The alpha power during the trial and baseline period was computed using 1 Hz resolution Morlet wavelets. The real and imaginary components were separately used to calculate the inverse for each set of values for each trial. To calculate the noise covariance matrix for the inverse calculation, the baseline data from 1 s prior to the start of the trial was mean subtracted on a trial by trail basis. The noise covariance was calculated for each trial and the final matrix was the mean of all artifact free trial matrices for each specific block. To increase the robustness of the solution, we assumed covariance between channels was zero and used the diagonal of the matrix. The orientation of dipoles on the cortical surface were constrained perpendicular to the surface under the assumption that the primary source of the EEG is coherent postsynaptic potentials across populations of pyramidal neurons that are arranged perpendicular to the cortical surface (Buzsáki et al., 2012).

### ROI selection

Our ROI selection method utilized a pipeline similar to our previous work (Yuan et al., 2008). The time course of each electrode was transformed into its time-frequency representation using a 1 Hz band Morlet wavelet and the power in each time window and frequency band (from 1 to 50 Hz) was computed (Qin and He, 2005). Mean amplitude at each sensor in the alpha band (8–13 Hz) was calculated with the real and imaginary parts. Source imaging was then performed with the real and imaginary parts separately to obtain the corresponding CCD amplitudes which were then combined to compute a total frequency-specific CCD.

**Figure 1:**
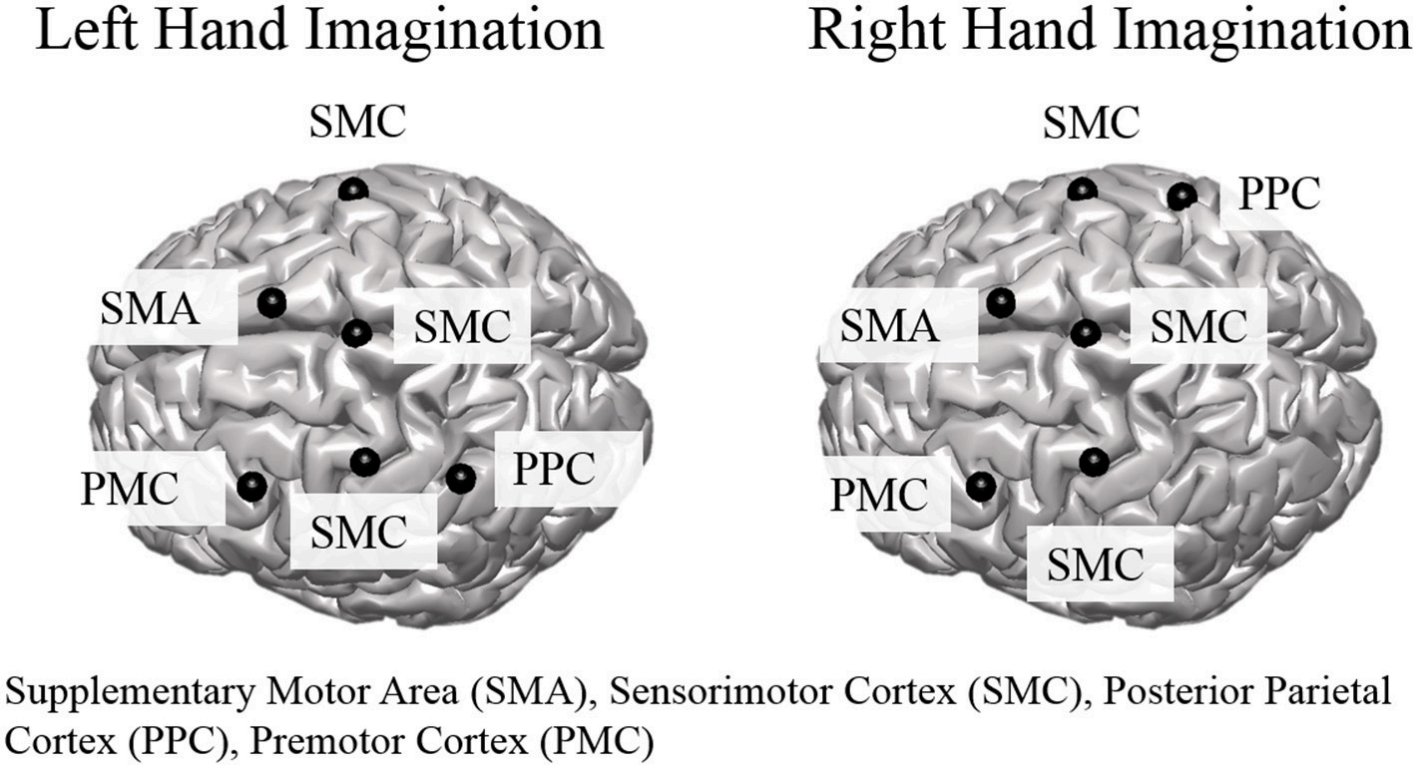
Group level regions of interest for left and right hand imagination. Black sphere indicates the center of the ROI. SMC, Sensorimotor Cortex; SMA, supplementary motor area; PMC, premotor cortex; PPC, posterior parietal cortex.

Group level ROI selection was performed iteratively based on the mean CCD across all subjects for all sessions. First, all dipoles were assigned an alpha-band score based on the mean CCD across subjects and sessions which was calculated at each dipole over the entire control period for each trial. The dipole with the largest alpha-band score was taken as the center of the first ROI. The extent of the ROI was taken as other dipoles within a 2 cm radius that had an alpha-band score of at least one-quarter of the center dipole. The alpha-band score of all dipoles within a 3 cm radius were then set to zero, and the largest alpha-band score of those remaining was selected and this proceeded iteratively until the top 10 ROIs were determined. ROIs to analyze further for connectivity were selected from the aforementioned set based on knowledge of active areas during MI and BCI task performance (Lotze and Halsband, 2006; Hétu et al., 2013) and were limited to the frontal and parietal cortices. ROI were determined separately for left and right hand imagination (Figure 1). For both left and right hand imagination the center of the ROIs were located in 1. Right sensorimotor cortex (SMC), encompassing sections of the premotor, primary motor, and S1 cortices; 2. Left premotor cortex (PMC); 3 SMA, encompassing sections of the SMA bilaterally; 4. Left SMC, encompassing sections of the premotor, motor, and S1 cortex; 5. Bilateral midline SMC. In addition, for left hand imagination, the left PPC was included whereas for right hand imagination the right PPC was included.

**Figure 2:**
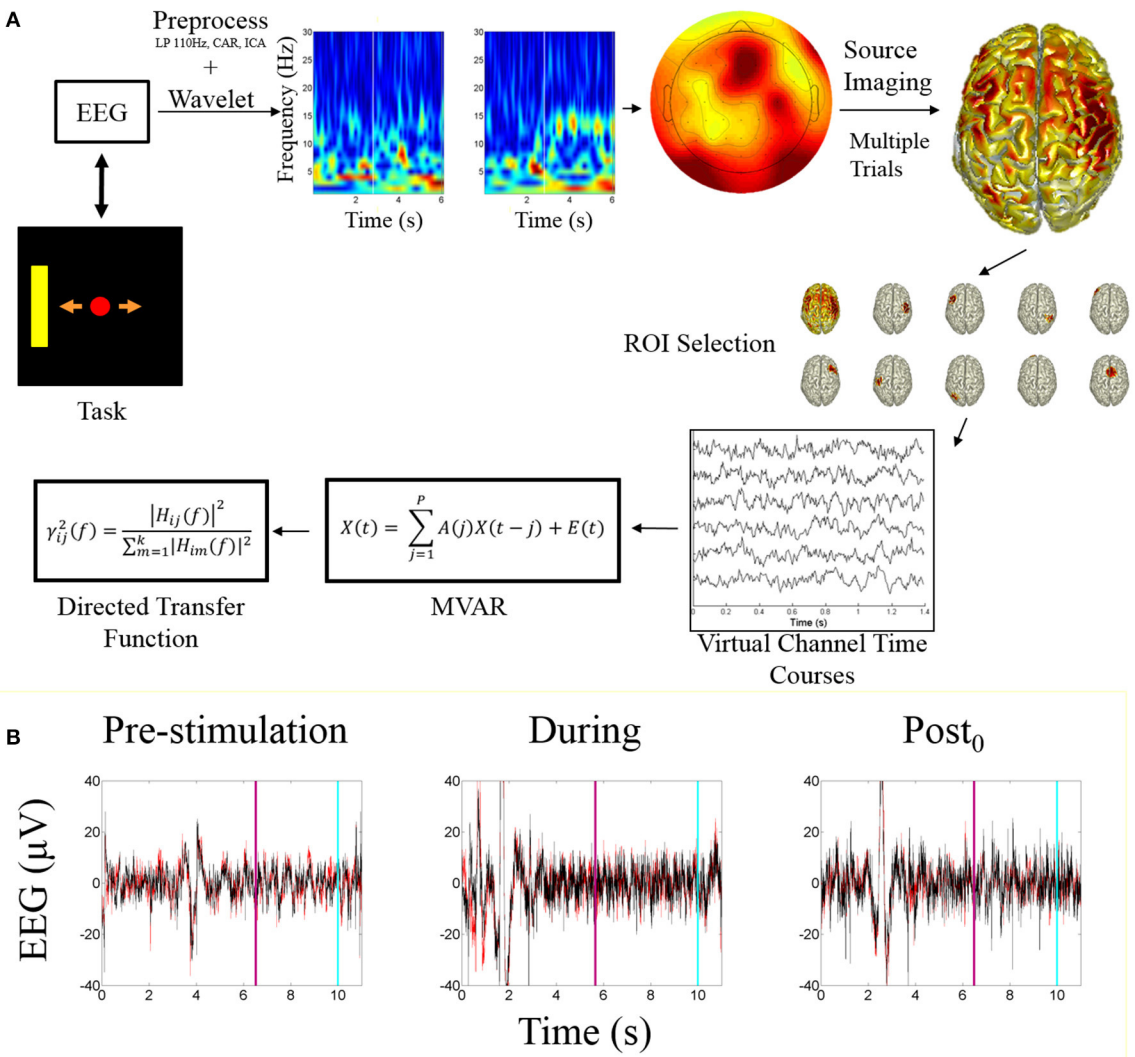
Processing pipeline and EEG data**. (A)** Data analysis processing pipeline. The starting point is the task visualization which is controlled by the EEG data. The included time-frequency transform is an example of a correct trial of right hand imagination, the white bar indicates when online feedback began. The sensor level topograph illustrates alpha band activity during an example trial. The source imaging distribution illustrates the mean alpha power distribution across all subjects for right hand imagination. ROI selection is performed based on the identification of the ROIs described within the text. The peak ROIs are found for each subject each session and the trial specific time courses are extracted and fit with a multivariate autoregressive model to which the directed transfer function is applied. **(B)** Example EEG time course from before, during, and after stimulation. Red trace is C3; Black is C4. Light blue line represents when the trial ended; purple line indicates when the feedback period began.

Subject specific ROIs on a session by session basis were determined by calculating the highest alpha-band scores across cortical dipoles for each subject within each session, within each of the global ROIs. The ROI activity time course was calculated by taking the mean of the dipoles within a 5 mm radius around the peak dipole. These time courses were used as a source-based virtual channels for analysis. These virtual channels were utilized for fitting the multivariate autoregressive model (MVAR) followed by analysis using the directed transfer function. An overview of the processing pipeline is illustrated in Figure 2A.

### Connectivity analysis

The multivariate autoregressive model is defined by

$$X\left(t\right)+\;\sum_{j=1}^{P}A\left(j\right)X\left(t-j\right)\;=\;E(t)$$

Where $X\left(t\right)={\left[X_{1}\left(t\right),X_{2}\left(t\right),\ldots X_{k}\left(t\right)\right]}^{T}\;$are the *k* time series at time *t* and $E\left(t\right)={\left[E_{1}\left(t\right),E_{2}\left(t\right),\ldots E_{k}\left(t\right)\right]}^{T}$ are the *k* white noise values at time *t*, and $A\left(j\right)=\;\left(\begin{array}{c} A_{11}\left(j\right)\ldots \;A_{1k}\left(j\right)\\ A_{k1}\left(j\right)\ldots \;A_{kk}\left(j\right) \end{array}\right)$ for *j* = 1,…,*p* are model parameters derived from the data.

*E(t)* is the uncorrelated white noise input driving the system with zero mean. The number of channels, *k*, was determined based on the number of ROIs chosen for connectivity. Model order, *P*, was determined using the AIC with the ARfit toolbox (Schneider and Neumaier, 2001) with each trial in each block being independently fit, then the mean of all trials per block taken as the order for all trials in that block, and each trial refit using the specified model order for that block. For most trials, cross and autocorrelation across 20 time lags exceeded the 2/sqrt(Nt) threshold, where Nt indicates the number of time points in the analysis window, which is a measure of the goodness of fit of the MVAR model, <10% of the time (Ding et al., 2000).

The directed transfer function calculates the connectivity between regions of interest for each frequency of interest. The directed transfer function evaluates the directed influence from one channel to another based on MVAR model fit to the data (Kaminski and Blinowska, 1991; Kaminski et al., 2001).

X(f)=H(f)E(f)

Where *H* is the transfer matrix defined in the frequency domain as [where A(0) is the identity matrix].

$$H\left(f\right)=\;{\left(\sum_{m=0}^{p}A(m)e^{-2\pi imf\Delta t}\right)}^{-1}$$

This can then be normalized to the total inflow to each channel yielding the normalized directed transfer function.

$$\gamma_{ij}^{2}\left(f\right)=\frac{{\left|H_{ij}(f)\right|}^{2}}{\sum_{m=1}^{k}{\left|H_{im}(f)\right|}^{2}}$$

### Statistical analysis

All statistics were performed in R. In order to evaluate changes in connectivity following stimulation, we subtracted the pre-stimulation connectivity values from the Post_0_ and Post_25_ connectivity values to calculate mean difference values. We applied a general linear model using the nlme package with fixed effects of condition (anode or sham), and random effects of session nested within each subject. The Shapiro test was used to evaluate the Gaussianity of connectivity values and the residuals of the model fit, if Gaussianity was significantly rejected for both of these measures (*p* < 0.05) non-parametric statistical tests (Wilcoxon rank sum test) were used to compare subject mean post-stimulation values across conditions; two sample for between groups and one sample for change from baseline. All values reported in text are mean ± standard error. *p*-values are uncorrected unless otherwise indicated. Cohen's *d* effect sizes were computed between conditions on normally distributed data using the compute.es package.

A generalized linear model with the fixed effects of each connectivity value and random effect of subject with levels by session and block was used to examine the relationship between behavioral measures and connectivity across all subjects regardless of condition. A Poisson link function was used when analyzing the number of correct trials; for time to hit, a Gamma link function was used as this empirically fit the data well. *p*-values are uncorrected unless otherwise indicated.

## Results

**Figure 3:**
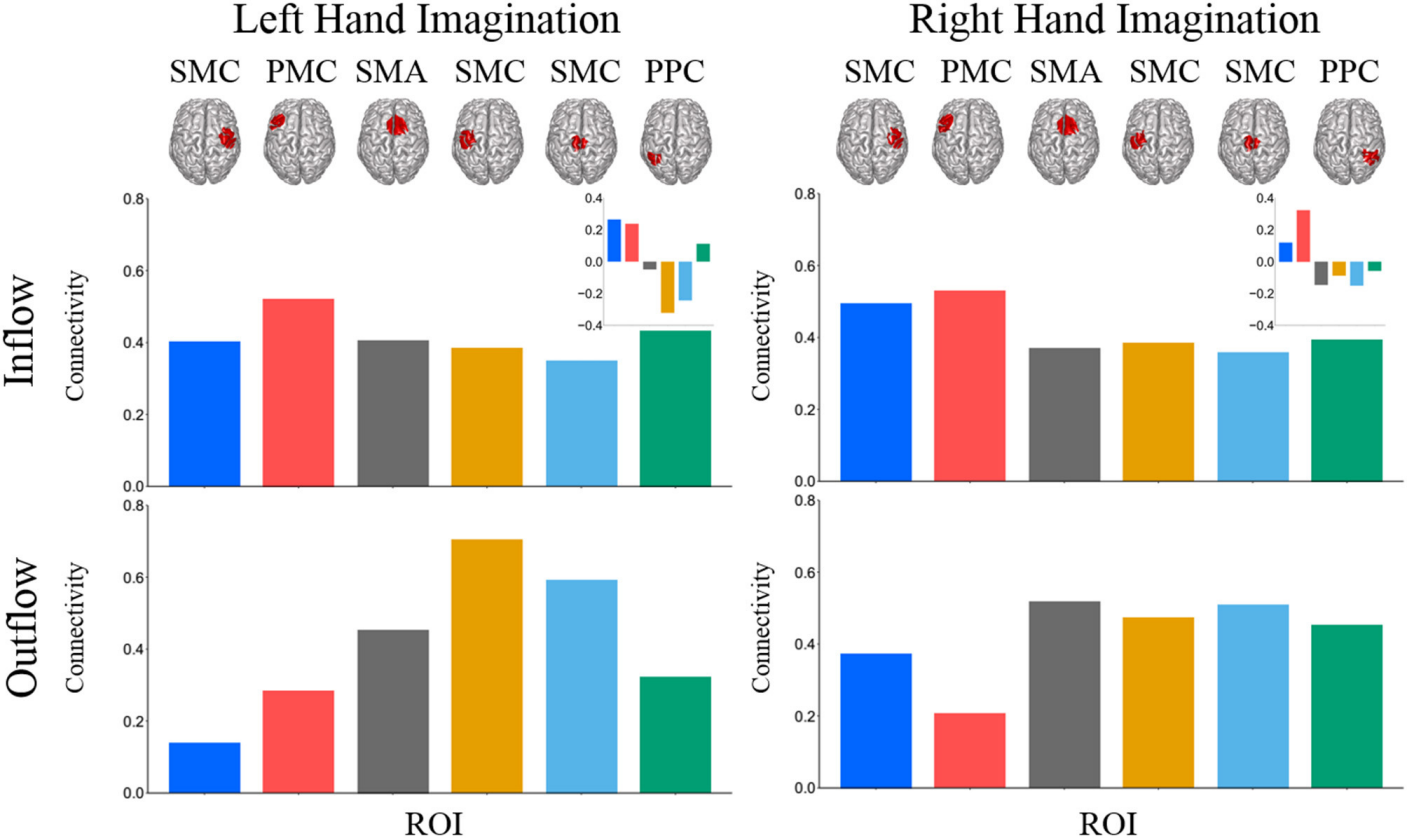
Mean total inflow and outflow across all subjects. Inset represents the difference between inflow and outflow for each ROI. For left hand imagination (Left) inflow to the left PMC is largest, while outflow from the left SMC is largest. The difference between inflow and outflow is most positive in the right SMC and left PMC, while the left SMC and midline SMC have the most negative difference. For right hand imagination (Right) the greatest inflow is to the left PMC, which also has the largest difference between inflow and outflow.

**Figure 4:**
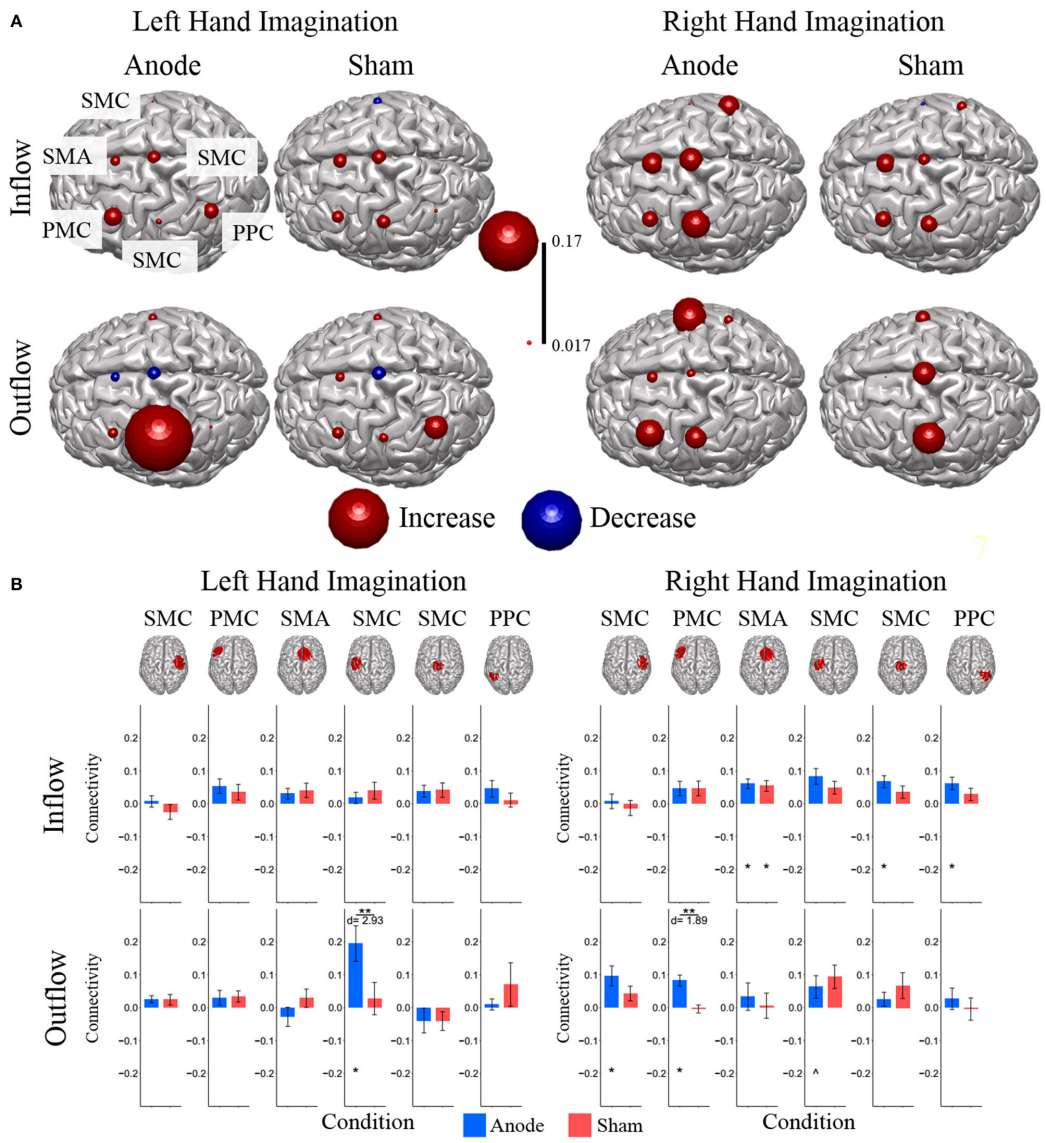
Alpha band normalized DTF total flow for each ROI**. (A)** Change in total connectivity from the pre-stimulation time point mapped onto the peak dipole of each ROI for anode and sham conditions. Color indicates the direction of change and sphere size indicates the absolute change value. **(B)** Mean post-stimulation change in total connectivity between conditions. For left hand imagination (Left) there was a difference in total outflow from the left sensorimotor cortex between groups, with a significant increase from pre-stimulation in the anodal group. For right hand imagination (Right) there was a significant difference in outflow from left PMC between groups. There were significant increases in the inflow to SMA, midline SMC, and right PPC; outflow from left SMC, left PMC, and right SMC in the anodal group and SMA inflow in the sham group. Total inflow to the ROI (top) and total outflow from the ROI (bottom). Bar color indicates the condition: anode (blue) and sham (red). Values are mean across subject; error bars are standard error across subjects. ^**^p < 0.05 between conditions. ^*^p < 0.05 change from pre-stimulation. ^∧^Wilcoxon rank sum change from pre-stimulation. *d* is Cohen's d effect size.

Composite inflow and outflow characterize the sum influence to and from each ROI (Figure 3). For left hand imagination inflow to the left PMC is largest, while outflow from the left SMC is largest. The difference between inflow and outflow is most positive in the right SMC and left PMC, while the left SMC and midline SMC have the most negative difference. For right hand imagination the greatest inflow is to the left PMC, which also has the largest difference between inflow and outflow. Within each target direction and condition, only a single ROI showed a significant difference in inflow or outflow between post-stimulation time points (Wilcoxon rank sum, *p* < 0.05 uncorrected) therefore the mean of these time points on a subject by subject basis was taken for further analyses. In general, there was a trend toward higher inflow and outflow at the Post_25_ time point compared to the Post_0_ time point. Total inflow to and outflow from ROIs in the alpha and beta bands changed based on laterality of hand imagination and stimulation condition as measured by the normalized DTF (Figure 4). For left-hand imagination there was a difference in total outflow from the left sensorimotor cortex between groups (*p* = 00048; *d* = 2.93), with a significant increase from pre-stimulation in the anodal group (0.19 ± 0.05; *p* = 0.016).

For right-hand imagination there was a significant difference in outflow from left PMC between groups (*p* = 0.0084; *d* = 1.89). There were significant increases in connectivity values in the anodal group across multiple areas of interest including inflow to SMA (0.06 ± 0.015; *p* = 0.003), midline SMC (0.068 ± 0.018; *p* = 0.006), and right PPC (0.062 ± 0.019; *p* = 0.020) and outflow from left SMC (0.063 ± 0.034; *p* = 0.031), left PM (0.082 ± 0.017; *p* = 0.011), and right SMC (0.097 ± 0.030; *p* = 0.028) and in the sham group in SMA inflow (0.054 ± 0.016; *p* = 0.02).

**Figure 5:**
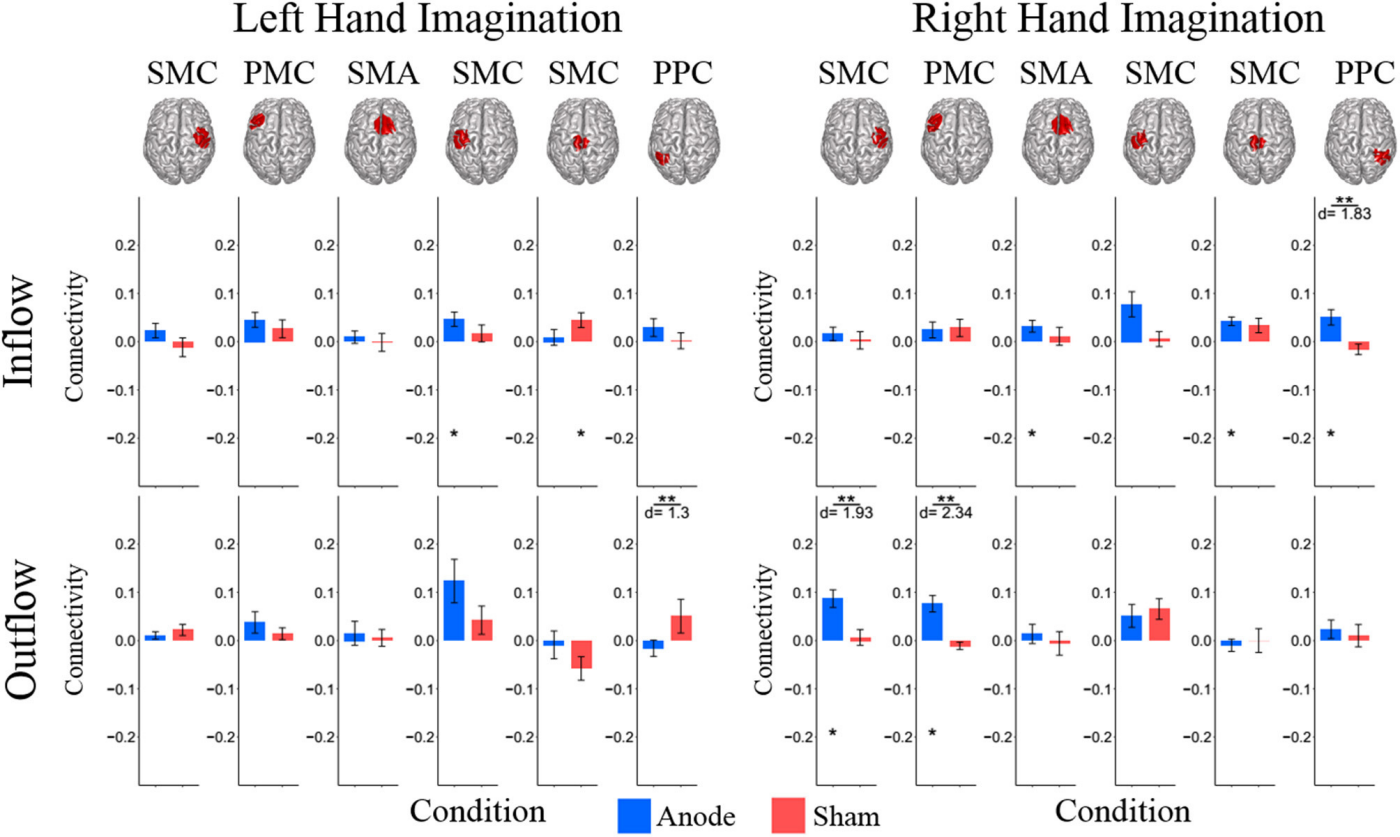
Beta band normalized DTF total flow for each ROI. Mean post-stimulation change in total connectivity between conditions. For left hand imagination (Left) there was a difference between conditions of outflow from the left PPC. There was a significant increase in inflow to the left SMC following anodal stimulation, and a significant increase to the midline SMC in the sham group. For right hand imagination (Right) there was a significant difference in inflow to the right PPC between groups and outflow from the right SMC and left PMC. There was an increase in SMA, midline SMC, and left PPC inflow and right SMC and left PM outflow following anodal stimulation. Total inflow to the ROI (top) and total outflow from the ROI (bottom). Bar color indicates the condition: anode (blue) and sham (red). Values are mean across subjects; error bars are standard error across subjects. ^**^p < 0.05 between conditions. ^*^p < 0.05 change from pre-stimulation. *d* is Cohen's d effect size.

In the beta band there were similar changes from pre-stimulation as there were in the alpha band. There were significant differences in beta frequency band connectivity within and between conditions (Figure 5). For left-hand imagination there was a difference between conditions of outflow from the left PPC (*p* = 0.048; *d* = 1.30). There was a significant increase in inflow to the left SMC following anodal stimulation (046 ± 0.015; *p* = 0.025), and a significant increase to the midline SMC in the sham group (0.045 ± 0.015; *p* = 0.043). For right-hand imagination there was a significant difference in inflow to the right PPC between groups (*p* = 0.0097; *d* = 1.83) and outflow from the right SMC (*p* = 0.0075; *d* = 1.93) and left PMC (*p* = 0.0023; *d* = 2.34). There was an increase in SMA (0.032 ± 0.012; *p* = 0.044), midline SMC (0.042 ± 0.009; *p* = 0.0023), and right PPC (0.050 ± 0.016; *p* = 0.028) inflow and right SMC (0.087 ± 0.018; *p* = 0.0016) and left PMC (0.076 ± 0.017; *p* = 0.013) outflow following anodal stimulation.

**Figure 6:**
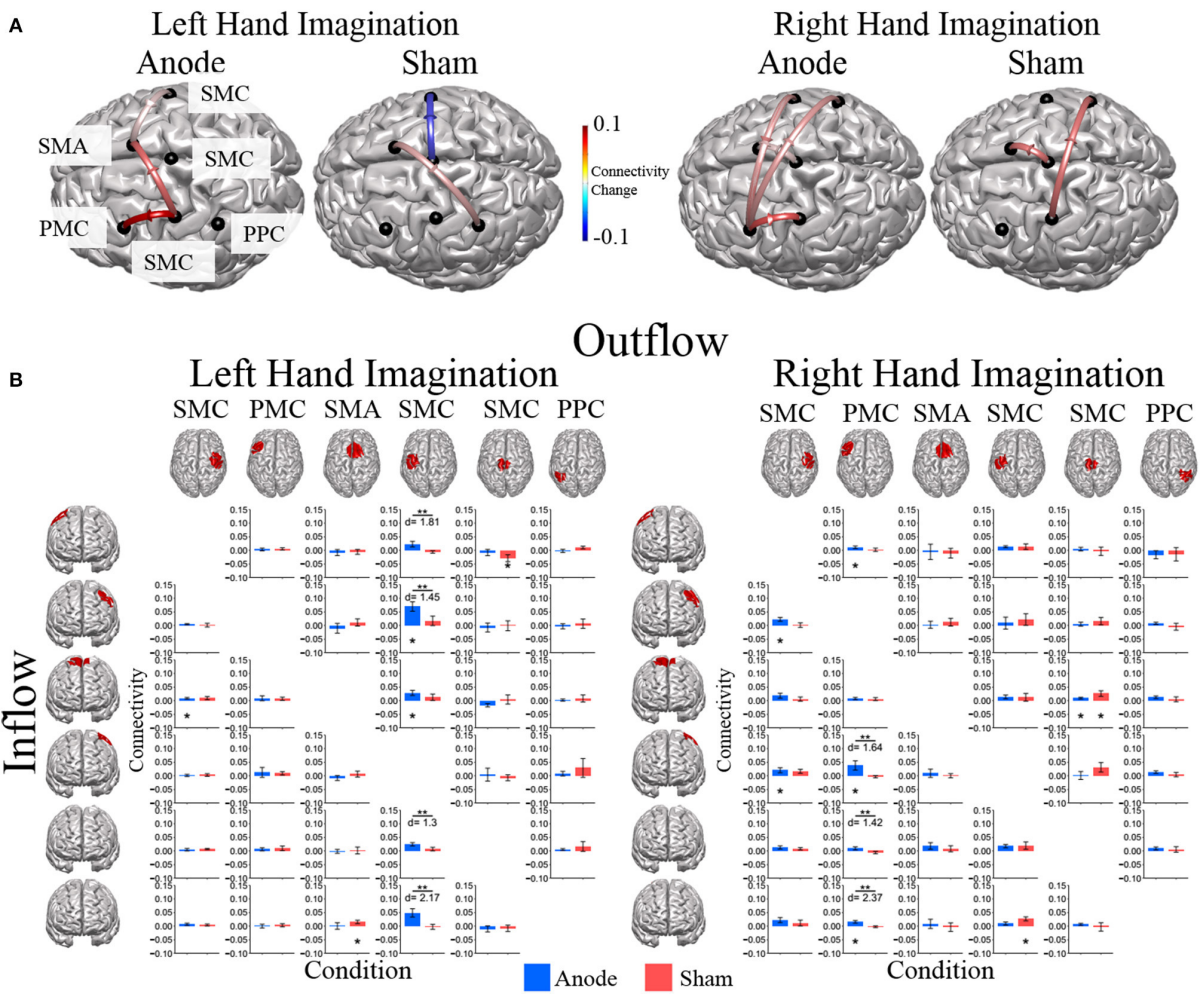
Alpha band normalized DTF flow between each ROI pair**. (A)** Alpha band changes in connectivity after stimulation for right and left hand imagination in the anode and sham stimulation groups. Red indicates and increase in connectivity, blue indicates a decrease in connectivity. Values are mean across subjects across blocks. All included connections had *p* < 0.05 from pre-stimulation baseline. **(B)** Mean post-stimulation change in directed connectivity between conditions. For left hand imagination (Left) there were significant differences between groups for output from the left SMC to right SMC, left PMC, midline SMC, and left PPC, with the anodal group having a greater increase than the sham group. The anodal group had increased flow from right PMC to SMA, left SMC to left PMC and SMA. The sham group had increased connectivity from SMA to right PPC and midline SMC to right SMC. For right hand imagination (Right) there were differences between groups from left PM to left SMC, midline SMC, and right PPC with higher changes in connectivity in the anodal group. For the anodal group there was increased connectivity from right SMC to left PM and left SMC, from left PM to right PMC, left PMC, and right PPC, from midline SMC to SMA. For the sham group increased connectivity from left SMC to right PPC and midline SMC to SMA. Values are mean across subjects; error bars are standard error. ^**^p < 0.05 between conditions. ^*^p < 0.05 change from pre-stimulation. *d* is Cohen's d effect size.

Directed connections between the ROIs in the alpha band display further differences due to HD-tDCS based on the laterality of hand imagination (Figure 6). For left-hand imagination there were significant differences between groups for output from the left SMC to right SMC (*p* = 0.011; *d* = 1.81), left PMC (*p* = 0.031; *d* = 1.45), midline SMC (*p* = 0.048; *d* = 1.30), and left PPC (*p* = 0.0037; *d* = 2.17), with the anodal group having a greater increase than the sham group. The anodal group had increased flow from right SMC to SMA (0.0086 ± 0.0030; *p* = 0.010) and left SMC to right PMC (0.070 ± 0.017; *p* = 0.0084) and SMA (0.029 ± 0.009; *p* = 0.028). The sham group had increased connectivity from SMA to right PPC (0.015 ± 0.0065; *p* = 0.043) and midline SMC to right SMC (−0.029 ± 0.013; *p* = 0.036).

For right-hand imagination there were differences between groups from left PMC to left SMC (*p* = 0.037; *d* = 1.64), midline SMC (*p* = 0.033; *d* = 1.42), and right PPC (*p* = 0.002; *d* = 2.37) with higher changes in connectivity in the anodal group. For the anodal group there was increased connectivity from right SMC to left PMC (0.023 ± 0.007; *p* = 0.021) and left SMC (0.021 ± 0.009; *p* = 0.043), from left PMC to right PMC (0.011 ± 0.005; *p* = 0.03), left SMC (0.038 ± 0.017; *p* = 0.037), and right PPC (0.016 ± 0.005; *p* = 0.0039), and from midline SMC to SMA (0.009 ± 0.004; *p* = 0.043). For the sham group increased connectivity from left SMC to right PPC (0.027 ± 0.007; *p* = 0.023), and midline SMC to SMA (0.026 ± 0.010; *p* = 0.021).

**Figure 7:**
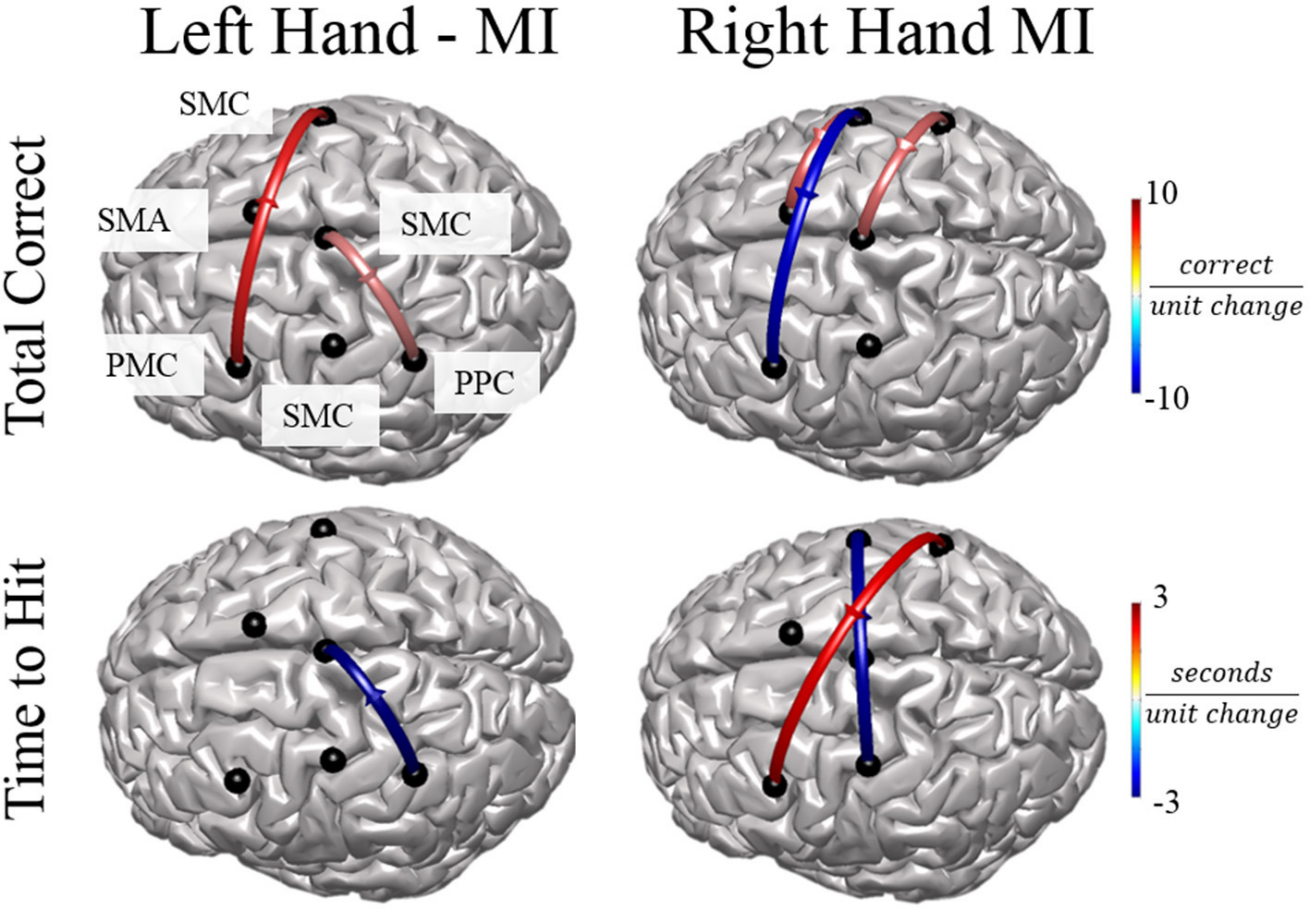
Connectivity Value–Behavioral Correlations. For both right and left hand imaginations, flow from the ipsilateral PPC to midline SMC correlated with improved performance. For right hand imagination, inflow to left PMC, in particular from right SMC and PPC correlates with reduced performance through reduced total correct and increased time to hit. Flow from left SMC to right SMC and SMA correlates with improved performance through an increased total correct and decreased time to hit. For left hand imagination, flow from left PMC to right SMC and flow from midline SMC to left PPC correlated with improved performance. Color indicates the beta coefficient value *p* < 0.05 for all displayed connections.

In order to examine the relationship between alpha band connectivity values and behavioral performance metrics we utilized a generalized linear model with each normalized connectivity value as a fixed effect within the same model. Behavioral metrics across all subjects were: Mean time to hit correct targets for right-hand trials (RH) was 3.511 ± 0.337 s and left-hand trials (LH) was 3.718 ± 198.64 s. Mean correct targets per block were RH: 7.88 ± 1.75 trials and LH: 8.52 ± 2.09 trials. Overall, specific normalized connectivity values were correlated with behavioral outcome measures (Figure 7). For right-hand imagination trials, multiple connections correlated with decreased performance, in particular, total inflow to right PMC was significantly correlated with an increased time to hit (β = 7910, *p* = 0.039). Flow from right SMC to left PMC correlated with a decreased total correct (β = −6.24, *p* = 0.003, *p* < 0.048 FDR corrected). Flow from right PPC to left PMC correlated with an increased time to hit (β = 1729, *p* < 0.018). However, other connections correlated with improvements in behavioral measures. Flow from left SMC to right SMC correlated with a decreased time to hit (β = −2478, *p* < 0.027). Flow from left SMC to SMA correlated with an increased total correct (β = 3.22, *p* = 0.022). Flow from right PPC to midline SMC correlated with an increased total correct (β = 2.96, *p* = 0.031). For left-hand imagination trials we found correlations between directed connections and improved behavioral metrics. Flow from left PMC to right SMC correlated with an increased total correct (β = 4.78, *p* < 0.023). Flow from left PPC to midline SMC correlated with a decreased time to hit (β = −2,955, *p* = 0.007). Flow from midline SMC to left PPC correlated with an increased total correct (β = 2.33, *p* = 0.019).

## Discussion

Unilateral high-definition anodal tDCS during motor imagery-based brain computer interface performance has bilateral connectivity effects. Stimulation aftereffects differ based on the laterality of hand imagination, with an increased effect on connectivity when performing right-hand imagination, contralateral to the stimulated hemisphere. These results suggest that tDCS interacts with ongoing task-specific endogenous oscillations and affects communication between brain areas involved in task performance. To the best of our knowledge, this is the first study examining connectivity changes following tDCS and correlating connectivity to behavioral performance to examine how planned targeting of the network with stimulation could be used to improve performance.

### Motor network connectivity

During right-hand imagination, the left sensorimotor cortex desynchronizes (and vice versa for left-hand imagination), which is characterized by a relative decrease in power in the alpha band. This decrease in power is due to networks within the sensorimotor cortex altering their firing patterns when activated by the MI task, this is referred to as event-related desynchronization (ERD) (Pfurtscheller and Lopes da Silva, 1999). The directed connectivity of MI during BCI varies based on the laterality of imagination and there is a large degree of interconnectivity within the sensorimotor cortex bilaterally, including the premotor, motor, and parietal cortices (Gao et al., 2011). These differences and connectivity patterns are likely due to event related synchronization and desynchronization across the motor cortex and the interactions with the rest of the motor imagery network through cortico-cortical connections both intrahemispherically and across the corpus callosum between the two hemispheres connecting the motor and parietal cortices. Gao et al. directly compared directed network activity during ME and MI and found similar connections in both, with ME having multiple significantly stronger connections (Gao et al., 2011). For right-hand imagination, we found greater inflow than outflow in the left PMC and right SMC, with greater outflow than inflow in the SMA and left SMC, and similar inflow and outflow in the right PPC. For left-hand imagination, we found greater inflow than outflow in the right SMC and left PMC, with greater outflow than inflow in the left SMC and midline SMC. Our results differ in relative connectivity when examining the normalized inflow and outflow compared to the work of Gao and colleagues. A reason for this may be based on the composition of the network, where Gao examine more regions across the cortex, which may offset this balance of inflow and outflow. In addition, we report frequency specific connectivity where previous work examined non-frequency specific BOLD activity.

Specific directed connections have also been investigated during MI and execution. Athanasiou et al. examined connectivity in the alpha band during MI and found information flow from contralateral to ipsilateral M1 and SMA to ipsilateral M1 (Athanasiou et al., 2012). We report connectivity in these directions, though they are not the connections of greatest strength. Anwar et al found a high degree of effective connectivity from SMC to PMC as well as bidirectional SMC–PMC connectivity during ME task performance in the hemisphere contralateral to the hand movement. We find a similarly high connectivity during right hand imagination, from SMC to PMC in the contralateral hemisphere. An important difference in the experimental design of this study was the examination of connectivity as subjects received feedback during imagination through the BCI task. This may account for the differences within the motor network as well as there is constant evaluation of performance and movement which is not present or examined in these previous studies.

### Effect of tDCS on motor network activity

Polania et al examined undirected connectivity at the EEG sensor level using graph theory measures and found increased connectivity in the alpha band within the left hemisphere during a right hand motor task following anodal stimulation over the left primary motor cortex (Polanía et al., 2011). During right hand imagination we found increased directed connectivity within the left hemisphere, specifically from the PMC to SMC, which suggests that the undirected connections found by Polania are specific to this direction. In addition, Polania and colleagues found no change or decreased interhemispheric connectivity based on specific electrodes in the alpha band following stimulation, whereas we report an increase in connectivity from the left PMC to the right SMC and right PPC as well as from the right SMC to the left PMC and left PMC. Polania and colleagues used resting state fMRI to examine cortico-thalamic connectivity following anodal tDCS and found increased connectivity between the stimulated left primary motor cortex, and subcortical structures of the ipsilateral thalamus and caudate nucleus (Polanía et al., 2012). As the thalamus has widespread cortico-cortico connections, this is a possible pathway through which the intrahemispheric changes we report occur, though the use of EEG precludes the analysis of connectivity including deep thalamic sources and interhemispheric corpus callosum pathways which in reality may contribute to these changes.

Notturno and colleagues examined functional connectivity using EEG during ME following tDCS with the anodal electrode over the left SMC and found no effect on coherence, an undirected measure, during motor movement between C3 and any other electrode following anodal or cathodal stimulation, though they did not look at pairwise coherence between other electrodes (Notturno et al., 2014). Our results suggest significantly different effects of stimulation in a directed manner. An explanation for the previously found lack of effect by Notturno and colleagues may be the relationship between the timing of stimulation and task performance. These previous studies applied stimulation during rest whereas we had subjects perform the task concurrent with stimulation. During sensorimotor rhythm modulation for controlling a BCI, the control signal is generated from both hemispheres whereby there may be increased interaction between the two during task performance. Combining task performance with stimulation may then increase both ipsilateral and contralateral connectivity due to task specific activity. These differences highlight the importance of context with stimulation, whereby differing activity during stimulation leads to differing aftereffects of the stimulation on task specific activity, as has been previously suggested (Buch et al., 2017). These differences may also be partially explained by the fact that subjects performed ME in this previous study, even though the motor network connectivity is quite similar between these two activities, as described previously.

A limitation of the current study is that although we instructed subjects to perform kinesthetic imagination of the left and right hand, subjects may have adapted their imagination to improve performance, by using a more complex MI that experimentally allowed them better control of the cursor; for example, throwing a ball or opening a door handle. In order to improve our understanding of the relationship between connectivity and performance, analyses of the connectivity over the time-course of individual trials is needed. Another limitation of this work is the number of subjects used for the analysis. As this was an initial exploratory analysis of the effects of stimulation on connectivity, the number of subjects, and the number of directed connectivity measures, are small and we did not correct our statistics for multiple comparisons. Further studies could utilize more subjects to examine the reproducibility of these analyses and examine specific directional connections based on a priori hypotheses to reduce the number of comparisons.

### Connectivity–behavior relationship

We found specific connectivity features that correlated with the changes in performance as measured by the number of correct trials and the time to hit correct targets. We do not attempt to predict performance based on these connectivity measures but rather use these correlations to examine how the network interacts during BCI performance. For both right- and left-hand imagination, flow from the ipsilateral PPC to midline SMC correlated with improved performance. The PPC is connected to the SMC and directs attention and visuomotor planning during ME and imagination (Lotze and Halsband, 2006). Generally, there is a slight increase in alpha power in the midline SMC during either right- or left-hand imagination as it is involved in lower limb movement rather than hand movement. This planning input from the ipsilateral PPC may effect this increase in alpha power, which in turn could lead to improved unilateral hand imagination through inhibition of midline SMC.

Right- and left-hand imagination also had differential connections correlated with performance. For right-hand imagination inflow to left PMC, in particular from right SMC and PPC correlated with reduced performance through reduced total correct and increased time to hit. As the left PMC is used in planning of both left and right hand movements, input from the right motor cortex may impair the planning functionality. In addition, the right motor cortex synchronizes during right-hand imagination whereby output may result in an inhibition of relevant information transfer from PMC to downstream regions. There were also connections that correlated with improved performance for right- and left-hand imagination. Flow from left SMC to right SMC and SMA correlates with improved performance through an increased total correct and decreased time to hit. During right-hand execution and imagination left SMC is desynchronized and is active in directing the movement or imagination. This output to the contralateral SMC may be an inhibitory signal via the corpus callosum, a known direct interhemispheric pathway (van der Knaap and van der Ham, 2011). During left hand imagination, flow from left PMC to right SMC correlated with improved performance. As the left PMC directs bilateral motor planning and as the right PMC is primarily active during left MI, increased information flow in this direction may improve performance. Our findings suggest that increases in the performance of behavioral measures are positively correlated with connections from planning regions, such as PPC and PMC, to sensorimotor cortex whereas a decreases in performance of behavioral measures correlate with connections from the SMC to planning regions.

To the best of our knowledge, there are no comparative works examining the correlation between connectivity and performance of BCI tasks. Though Billinger et al. (2013) examined offline classification performance of EEG source and sensor activity and connectivity features, they did not report specific connectivity features used for classification so we are unable to make comparisons with the current study. In addition, they did not relate connectivity to any online performance metrics but rather examined if connectivity based classifiers can be used to improve classification accuracy.

## Conclusion

Our results support the hypothesis that tDCS interacts with ongoing endogenous brain oscillations in an activity and task-specific manner. During motor imagery there is a decrease in alpha power in the contralateral sensorimotor cortex due to a desynchronization in the underlying networks, with areas involved in the imagination decoupling from surrounding areas. Based on unilateral sensorimotor stimulation, we see differing interactions of the stimulation aftereffect on network connectivity based on the laterality of hand imagination. We also show both positive and negative correlations between specific directed connection strengths and behavioral metrics, with connections from ipsilateral PPC to midline SMC correlating with behavioral improvements for both right and left hand imagination. However, HD-tDCS over the left SMC did not alter any of these connections that correlate with behavior. The effects of targeting network connections and the most efficacious methodology to alter networks using TCS is still unclear. Future work should examine targeting regions of interest with anodal stimulation to increase excitation and therefore increase the probability of correlating the firing in these areas, however the timing of the firing also needs to be considered as the directional effect of plasticity varies based on these correlations (Müller-Dahlhaus and Ziemann, 2015). Additional work using adaptive or short time directed transfer function, connectivity, and behavioral output to examine how these networks develop across time, will be vital for developing adaptive stimulation.

## Ethics statement

This study was carried out in accordance with the recommendations of the University of Minnesota Institutional Review Board with written informed consent from all subjects. All subjects gave written informed consent in accordance with the Declaration of Helsinki. The protocol was approved by the University of Minnesota Institutional Review Board.

## Author contributions

BB and BH designed the experiments. BB performed experiments. BE assisted in data collection. BB analyzed the data. All authors discussed the results and interpretations. BB and BH wrote the article.

### Conflict of interest statement

The authors declare that the research was conducted in the absence of any commercial or financial relationships that could be construed as a potential conflict of interest.
